# MiR-223 as a Regulator and Therapeutic Target in Liver Diseases

**DOI:** 10.3389/fimmu.2022.860661

**Published:** 2022-03-16

**Authors:** Jiarong Gu, Hao Xu, Yandong Chen, Na Li, Xin Hou

**Affiliations:** ^1^ School of Medicine, Ningbo University, Ningbo, China; ^2^ The Affiliated Hospital of Medical School, Ningbo University, Ningbo, China

**Keywords:** miR-223, macrophage, neutrophil, inflammation, liver disease

## Abstract

MicroRNAs (miRNAs) are endogenous non-coding single-stranded small molecule RNAs consisting of 20–24 nucleotides that are highly conserved in species evolution. Expression of miRNAs is strictly tissue-specific, and it is chronological in fungi and plants, as well as in animals. MiR-223 has been shown to play a key role in innate immunity, and dysregulation of its expression contributes to the pathogenesis of multiple inflammatory diseases, and cancers. In this article the biosynthesis and functions of miR-223 in innate immunity are reviewed, and the role of miR-223 in liver physiopathology and therapeutic prospects are highlighted.

## Introduction

In 1993, the microRNA (miRNA), Lin-4, was identified as a regulator of embryonic development in *C. elegans* (1). Since then, miRNAs have been an active area of a research. Ongoing work continues to elucidate the functions and regulatory networks of miRNAs, as well as their contributions to regulation of post-transcriptional gene expression (2). MiRNAs are endogenous non-coding, single-stranded small molecule RNAs that are 20-24 nucleotides in length and have been highly conserved over species evolution. The expression profiles of miRNAs are strictly tissue-specific, and are chronological in fungi, plants, and animals (3). One of the most authoritative databases currently available for miRNAs is miRBase, which includes both name and sequence information for miRNAs identified to date. The latest release of the miRBase database includes 38 589 hairpin precursors and 48 860 mature miRNAs from 271 organisms. These miRNAs regulate expression of at least 60% of protein-coding genes (4). At the post-transcriptional level, miRNAs regulate protein expression through complementary binding to 3′non-coding regions of target gene mRNAs to direct RNA-induced silencing complex-mediated degradation or to inhibit translation. As non-coding RNAs with important regulatory functions, miRNAs play important regulatory roles in normal physiological processes such as organism development, tissue remodeling, metabolism, immune responses, cell proliferation, cell differentiation, and intracellular signal transduction. Conversely, dysregulated miRNAs are associated with inflammatory diseases, metabolic abnormalities, and tumors (5).

Distinct expression profiles are observed for miRNAs from different cell types and organs, and this is consistent with the variety of functions they perform. For example, hepatic miRNAs play an important role in the pathogenesis of liver disease by regulating hepatic lipid metabolism, inflammatory injury, fibrosis, and tumor progression. MiRNAs can also be released in extracellular vesicles (EVs) that function as messengers for communication between hepatocytes and immune cells, or between liver and other tissues (6).

Most recently, emerging evidences have demonstrated that miR-223 is essential for development and homeostasis of the immune system. It may also have an essential role in both inflammation disorders and various liver diseases. The current review discusses miR-223 biogenesis and the function of miR-223 during innate immunity, while also highlighting its role in liver diseases. Potential applications of miR-223 in disease diagnosis and treatment are presented as well.

## MiR-223 Biosynthesis and Function

### MiR-223 Biosynthesis

The miR-223 gene is located within the q12 locus of the X chromosome and is highly conserved during evolution (7). The long primary transcript of miR-223 (pri-miR-223) contains a hairpin structure in exon 3 of the non-coding transcript which primarily results in production of the miR-223-3p strand (hereafter referred to as miR-223 unless specified). A minor product, miR-223-5p, is also produced which is prone to degradation, yet has been shown to play roles in several diseases (8).

MiR-223 is highly expressed in the myeloid lineage and is regulated by myeloid-transcription factors such as PU.1, CCAAT enhancer-binding protein alpha (C/EBPα), CCAAT enhancer-binding protein beta (C/EBPβ), and Nuclear Factor I-A (NFIA). PU.1, C/EBPα, and C/EBPβ bind the promoter of miR-223 and increase its expression. C/EBPα can also cooperate with PU.1 to enhance miR-223 expression. In contrast, NFIA inhibits expression of miR-223. Interestingly, both NFIA and C/EBPβ are targets of miR-223, thus a negative feedback loops exists between miR-223 and its transcription factors (9, 10). Peroxisome proliferator-activated receptor gamma (PPARγ), a nuclear transcription factor, can enhance miR-223 expression by directly binding to PPARγ regulatory elements that are located in the promoter of miR-223 (11). Kruppel-like factor 6 (KLF6) is a unique member of the zinc-finger family of transcription factors. KLF6 represses miR-223 expression by occupying its promoter region, thereby promoting proinflammatory gene expression in macrophages (12). The factors that influence these transcription factors are also involved in miR-223 expression. For example, Sirtuin-1, an NAD^+^-dependent histone deacetylase, interacts with C/EBPα to induce its deacetylation, thereby inducing miR-223 expression in neutrophils (13). Meanwhile, macrophage colony-stimulating factor and receptor activator of nuclear factor kappa-B ligand can increase expression of PU.1 (14).

Epigenetic mechanisms, such as DNA methylation and post-translational modifications of nucleosomal histone proteins, contribute to miRNA regulation. In patients with acute myeloid leukemia (AML), AML1/ETO, a common AML-associated fusion protein, reduces miR-223 expression by recruiting a chromatin remodeling enzyme to the pre-miR-223 promoter binding site to silence heterochromatin of miR-223 (15). In atherosclerotic cerebral infarction patients, miR-223 levels have been found to negatively correlate with mean methylation levels of the miR-223 promoter, suggesting that miR-223 expression is inhibited by promoter hypermethylation (16). Furthermore, in hepatocellular carcinoma (HCC), miR-223 expression is suppressed by sulfatide, a sulfoglycolipid. The presence of sulfatide reduces recruitment of acetylated histone H3 and C/EBPα to the promoter of pre-miR-223, and this leads to miR-223 downregulation (17).

### Target Genes of MiR-223

**Figure 1:**
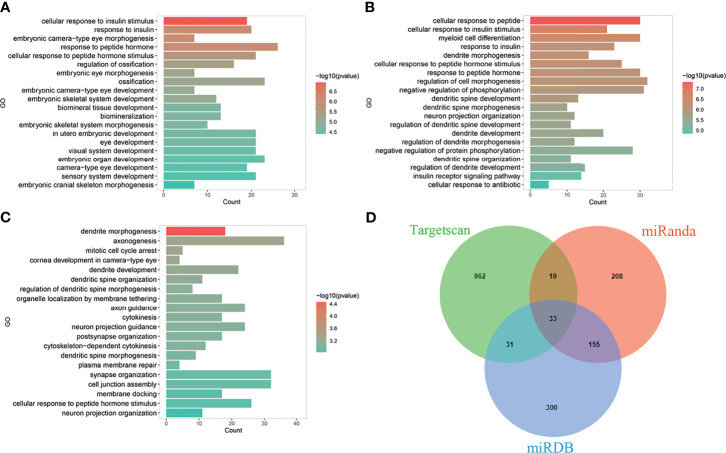
Biological function prediction of miR-223 target genes. Functions of miR-223 target genes analyzed in TargetScan database **(A)**, miRDB database **(B)**, and miRanda database **(C)**. **(D)** The intersecting target genes of the three databases.

**Figure 2:**
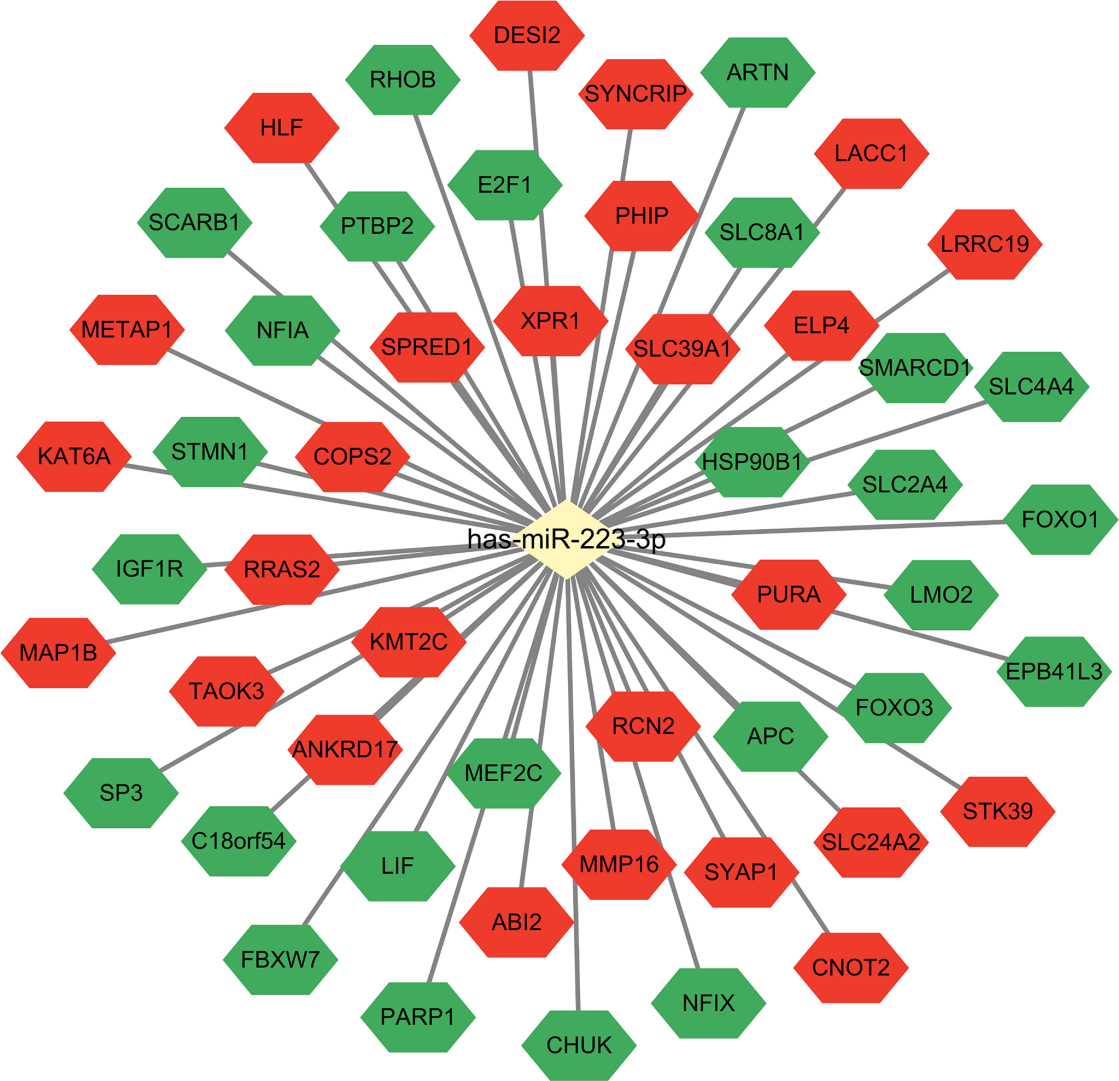
MiR-223 target genes. Combined with miRTarBase database, the target gene network of miR-223 was constructed. The green shapes represent experimentally validated target genes that have been reported, and the red shapes represent target genes that have not been experimentally validated yet.

The main function of miRNAs is to prevent expression of target genes *via* degradation or inhibition of mRNA translation. Since one miRNA may correspond to multiple target mRNAs. We performed bioinformatics analysis to predict target genes of miR-223. Several miRNA target genes databases were employed, including TargetScan (18), miRDB (19), and miRanda (20). The same databases were also used to perform functional enrichment analyses to identify biological functions of potential miR-223 target genes (**Figure 1**). The following biological processes were identified: cellular response to insulin stimulus, response to peptide hormone, regulation of ossification (TargetScan database); myeloid cell differentiation, regulation of cell morphogenesis, negative regulation of phosphorylation (miRDB database); and dendrite cell development and cytokinesis (miRanda database). While some of these miR-223 target genes have been reported, the others remain to be explored. There are newly identified target genes of miR-223 which are not included in **Figure 2** (e.g., Taz, Cxcl10, and Nlrp3), and these will be discussed below. Additionally, we added data from the experimentally validated database, miRTarBase (21) to construct a miR-223 target gene network (**Figure 2**). Within this network, 26 target genes have been confirmed experimentally (e.g., FOXO3, LMO2, KAT6A, etc.), while 26 are unreported (e.g., XPR1 and LACC1). It remains for future studies to elucidate what regulatory relationships may exist between these mRNAs and miR-223.

In summary, the gene encoding miR-223 is located within the q12 locus of chromosome X and its expression is enhanced by transcription factors, PU.1, C/EBPα, and PPARγ, and inhibited by NFIA and KLF6. Methylation and deacetylation also regulate miR-223 expression. MiR-223 has multiple target genes. We employed bioinformatics methods to predict new target genes, which may contribute to regulation of myeloid cell differentiation, insulin resistance, tumor cell proliferation or other processes. Additional experiments are needed to confirm and characterize the regulatory relationship of these target genes with miR-223.

## MiR-223 in Innate Immunity and Inflammation

### Contribution of MiR-223 to Regulation of Myeloid Differentiation

The role of miR-223 in granulocyte differentiation remains controversial. Upon retinoic acid treatment, C/EBPα replaces NFIA on the miR-223 promoter to increase miR-223 expression. While overexpression of miR-223 is sufficient to induce myeloid precursor cells to differentiate into granulocytes (9), myeloid-specific miR-223 negatively regulates granulocyte differentiation and activation. It has also been observed that miR-223 deficient mice (miR-223^-/y^) have an expanded granulocytic compartment due to an increased number of granulocyte progenitor cells (22). The same study further demonstrated that transcription factor, myocyte enhancer factor 2C (Mef2c) (another target of miR-223), promotes myeloid progenitor proliferation (22). Discrepancies among reported results may derive from use of distinct gene manipulation strategies and granulocytic lineages. Manipulates of miR-223 expression at defined granulocyte differentiation stages in future studies could help elucidate the multifarious roles of miR-223.

During the differentiation of monocytes to macrophages, expression of miR-223 is downregulated significantly. Repression of miR-223 expression leads to increased expression of a target gene inhibitor which represses expression of nuclear factor kappa-B kinase subunit alpha (IKKα) and inhibits NF-κB pathways (23). Granulocyte macrophage colony-stimulating factor-induced differentiation of monocytes and phorbol12-myristate 13-acetate (PMA)-induced differentiation of THP1 cells has also been abolished by a miR-223 inhibitor (24).

### MiR-223 Regulates Inflammation in Myeloid Cells

#### MiR-223 in Neutrophils

MiR-223 is the most abundant miRNA in neutrophils and it plays an important role in modulation of neutrophil maturation and activation. Correspondingly, miR-223^-/y^ mice are characterized by increased numbers of neutrophils in their bone marrow, peripheral blood, and lungs. The neutrophils miR-223^-/y^ mice are hypermature, and are hyperactive to stimulation. It has been demonstrated that miR-223 controls neutrophil recruitment to the lungs during *Mycobacterium tuberculosis* (Mtb) infection by directly inhibiting the expression of chemokines (CXCL2 and CCL3), as well as cytokine, IL-6, in myeloid cells. Furthermore, miR-223 modulates the release of TNF-α and IL-10 from Mtb-infected myeloid cells by reducing NF-κB activity (25). In agreement with Mtb infection, miR-223 suppresses IL-6 expression and subsequently suppresses p47^phox^ expression to alleviate production of reactive oxygen species (ROS) in ethanol-induced liver injury (26). Additionally, miR-223 suppresses NOD-like receptor family pyrin domain containing 3 (NLRP3) inflammasome activity through direct binding of the 3′ untranslated region of NLRP3, thereby reducing IL-1β expression in neutrophils (27).

#### MiR-223 in Macrophages

MiR-223 contributes to regulation of macrophage polarization and activation. For example, miR-223 facilitates the differentiation of macrophages into the alternative M2 phenotype in the pathophysiology of many diseases, including high fat diet (HFD)-induced adipose tissue inflammation (28), coxsackievirus B3 (CVB3)-induced viral myocarditis (29), wound repair (30), sepsis (31), liver inflammatory resolution (32), viral myocarditis (33), and multiple sclerosis (34). Mechanistically, miR-223 suppresses proinflammatory activation of macrophages, and induces alternative activation of M2 macrophages by inhibiting Pknox1 in adipose tissue inflammation, CVB3-induced viral myocarditis, and cutaneous wounds (28–30). Nfat5 and Rasa1 are direct targets of miR-223 and are critical for PPARγ or IL-4-induced miR-223-mediated alternative activation of macrophages in a HFD model and in sepsis (11). M2-polarized macrophages exhibit an anti-inflammatory phenotype and function, including phagocytosis, and promote resolution of inflammation and tissue repair (35).

In addition to regulation of macrophage polarization, miR-223 also inhibits macrophage inflammation responses to Toll-like receptor (TLR) ligand stimulation. TLR ligands decrease miR-223 expression in macrophages, and this increases expression of the miR-223 target gene, STAT3. STAT3 then enhances expression of the proinflammation cytokine IL-1β and IL-6 (36). Interestingly, IL-6 has been shown to promote macrophage inflammatory responses by down-regulating miR-223 (37). In another study, it was observed that the down regulation of miR-223 by TLR ligand promoted production of TNF-α, IL-6, and IL-1β *via* increased expression of Ras homolog gene family, member B (RhoB). RhoB activates both NF-κB and mitogen-activated protein kinase signaling (38). MiR-223 dampens inflammasome activation and IL-1β secretion in macrophages by targeting NLRP3 (39). MiR-223 also negatively regulates NF-κB activation in human monocytic cells by inhibiting p65 phosphorylation, which in turn leads to decreased expression of inflammatory cytokines (40). Additionally, miR-223 enhances matrix metalloproteinase expression by targeting the circadian rhythm protein, brain and muscle ARNT-like protein-1 (BMAL1), during Mtb infection (41).

In summary, miR-223 skews macrophages toward an anti-inflammatory phenotype by targeting Pknox1/Rasal/NFAT5 during inflammation. In various inflammatory disease models, macrophage inflammatory responses have been downregulated by miR-223 *via* targeting of STAT3, RhoB, and NLRP3 (35).

### MiR-223 in EVs and Intercellular Communication

EVs are membrane-bound, nanometer-sized vesicles that are released by cells under normal, stressed, or transformed conditions. EVs can be packaged with mRNA, non-coding RNAs, proteins, lipids, miRNAs, etc. MiRNAs can be released into extracellular space within EVs, particularly exosomes. When EVs containing miRNAs are taken up by neighboring cells or when they gain access to the circulation, the miRNAs are able to regulate target genes in recipient cells both locally and distally. MiR-223 is one of the predominant miRNAs that has been identified in EVs isolated from human peripheral blood (42). It is hypothesized that it is released from peripheral blood monocytes and neutrophils. It has also been reported that activated human macrophages release microvesicles to deliver miR-223 as a functional cargo into target cells such as monocytes, epithelial cells, fibroblasts, and endothelial cells. Furthermore, microvesicles from activated macrophages can induce the differentiation of naive monocytes into macrophages (43). Similarly, mesenchymal stem cells can skew macrophages to a M2 phenotype by transferring exosome-derived miR-223 (30).

During acute lung injury, neutrophils transfer miR-223 *via* EVs to lung epithelial cells and dampen acute lung injury through repression of poly (adenosine diphosphate-ribose) polymerase-1 (PARP-1) (44). On the other hand, exosomes containing miR-223 derived from vascular endothelial cells cooperate with exosomes containing miR-27b-3p derived from type II alveolar epithelial cells to regulate alveolar macrophage phenotypes by targeting regulator of G protein signaling-1 (RGS1) (45). Notably, when miR-223/142 mimics have been loaded into unstimulated microvesicles and delivered into murine lung tissue, macrophage activation and lung inflammation are suppressed *via* inhibition of NLRP3 inflammasome activity (46).

In the liver, hepatocytes express very low levels of pri-miR-223. In contrast, miR-223 levels are much higher in the hepatocytes of HFD-fed mice. This elevation is due to preferential uptake of miR-223-enriched EVs derived from neutrophils/macrophages. The selective transfer of miR-223 into hepatocytes is dependent on low-density lipoprotein receptors (LDLRs) in hepatocytes and on apolipoprotein E (APOE) in neutrophil-derived EVs (47). In addition, IL-6 signaling in myeloid cells promotes the transfer of miR-223-enriched exosomes to hepatocytes (48). Taken together, exosomal miR-223 is considered a source of new messengers for myeloid cells to communicate among themselves and with other cells.

In addition to EVs, lipoprotein particles such as high-density lipoprotein (HDL) are enriched with miR-223 in serum. MiR-223 is transported in plasma and delivered to recipient cells by HDL with functional targeting capabilities. Moreover, lipoprotein particles and EVs are easily distinguished. Thus, HDL particles have been purified which express ApoA-I, yet are negative for classic exosomal protein markers (e.g., CD63, HSP70, and ICAM-1) (49).

## MiR-223 in Liver Homeostasis

In the liver, miR-223 plays an important role in maintaining cholesterol homeostasis, hepatocyte apoptosis, lipolysis, and cell proliferation through negative regulation target genes. In particular, miR-223 directly or indirectly regulates three key processes that govern intracellular and systemic cholesterol levels: in the cholesterol synthesis phase, miR-223 directly targets and represses two cholesterol biosynthetic genes, 3-hydroxy-3-methylglutaryl-CoA synthase 1 (HMGCS1) and SC4MOL, to inhibit cholesterol biosynthesis. In the cholesterol uptake phase, miR-223 represses cholesterol uptake by controlling scavenger receptor class B member 1 (SCARB1, SR-BI), while miR-223 promotes cholesterol efflux by indirectly upregulating basolateral ATP binding cassette subfamily A member 1(ABCA1) in hepatocytes (50). Most recently, Zhao et al. have proposed a novel role for miR-223 in preventing lithogenic diet-induced cholesterol gallstone development. It is possible that miRNA-223 preferentially decreases cholesterol transportation from hepatocytes into the bile by directly targeting the biliary cholesterol transporters, ABCG5 and ABCG8 (51). Another recent study reported that the lipolytic gene diacylglycerol lipase alpha (DAGLA) is negatively regulated by miR-223, and this may be associated with lipolysis in the liver (52). Interestingly, under simulated microgravity, miR-223 is upregulated in rat liver tissue, thereby inhibiting hepatocyte proliferation *via* negative regulation of CDK2 and CUL1 (53).

Therefore, in summary, miR-223 regulates physiological functions of the liver by maintaining cholesterol homeostasis, hepatocyte apoptosis, lipolysis, and cell proliferation in hepatocytes. Correspondingly, dysregulation of miR-223 is associated with various liver diseases, including viral hepatitis, acute liver injury, alcoholic and non-alcoholic liver disease, cirrhosis, and HCC.

## MiR-223 in Liver Diseases

### MiR-223 in Viral Hepatitis

Infections caused by hepatitis B virus (HBV) and hepatitis C virus (HCV) infection can lead to acute and chronic hepatitis, cirrhosis, or HCC. Moreover, progression of each of these diseases is driven by sustained inflammation. To date, the roles of miR-223 in HBV and HCV infections remain to be elucidated due to a lack of suitable small animal models for HBV/HCV infections. However, evidence from clinical studies and *in vitro* cell culture experiments suggest that miR-223 may play an important role in controlling inflammation caused by virus infection. For example, in HBV-infected patients, serum levels of miR-223 are higher than in healthy controls. Similar levels of miR-223 have also been observed in patients with HCC, suggesting that miR-223 may represent a more general biomarker for liver injury, rather than a specific biomarker for HCC (54). However, it has been reported that miR-223 levels are downregulated in the serum of HBV-positive HCC patients compared to healthy controls. MiR-223 has also been found to be downregulated in HBx-transfected HepG2 cells and HepG2.2.15 cells, while its target gene, c-myc, is upregulated to enhance hepatocyte proliferation (55). In addition, circulating hepatitis B surface antigen particles can function as carriers of miRNAs, including miR-223 (56).

In fresh liver biopsies collected from HCV patients, miR-223 levels were significantly lower than those in normal liver tissues (57). Meanwhile, in a cohort study, higher levels of miR-223 were detected in the plasma of HCV patients exhibiting a sustained virologic response. In addition, miR-223 levels negatively correlated with liver injury scores (58). Taken together, these results suggest that miR-223 regulation is affected by HCV infection and treatment-based viral cures. Correspondingly, in patients co-infected with HCV and HIV, circulating miR-223 levels increased after treatment, possibly in association with reduced inflammation and NF-κB activation. Furthermore, these downstream effects have the potential to regulate other immune pathways associated with chronic liver inflammation and complications (59).

Thus, during viral infection, miR-223 may act as a negative regulator of immune regulation and inflammatory response, and therefore may represent a possible biomarker for viral hepatitis. However, the mechanistic details for miR-223 in hepatitis virus-induced inflammation and immune cell regulation remain to be characterized.

### MiR-223 in Acute Liver Injury

**Figure 3:**
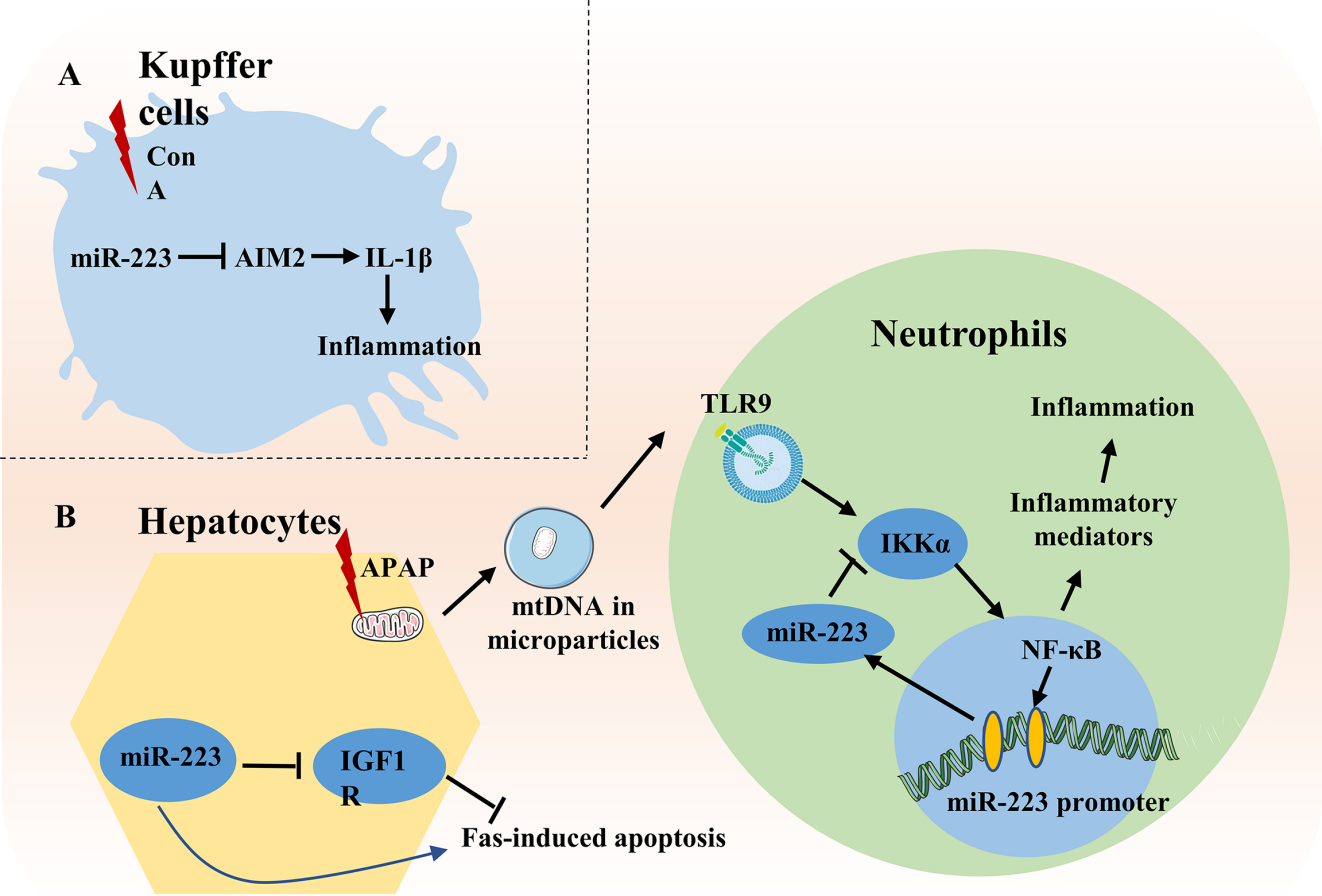
Role of miR-223 in acute liver injury. **(A)** In ConA-induced liver injury, miR-223 inhibits IL-1β production by suppressing inflammasome AIM2 in Kupffer cells. **(B)** During APAP-induced liver injury, mtDNA released from damaged hepatocytes activates NF-κB depending on TLR9-pathway and subsequently increases expression of inflammatory genes, thereby enhancing liver injury. Meanwhile, mtDNA/TLR9/NF-κB signaling also up-regulates expression of miR-223 in neutrophils. MiR-223 then acts as a negative feedback loop to ameliorate APAP-induced liver injury by targeting IKKα. During Fas-induced hepatocyte apoptosis, miR-223 enhances Fas-induced hepatocyte apoptosis and liver injury by targeting the insulin-like growth factor 1 receptor (IGF1R).

Acute liver failure is a rare and severe consequence of abrupt hepatocyte injury. It can evolve over days or weeks before reaching a lethal outcome. Substances that lead to hepatocyte injury can either directly cause toxic necrosis or induce apoptosis (within hours). Damaged hepatocytes release damage-associated molecular patterns to activate immune cells and evoke immune-based injury (which can occur over days to weeks). The most common cause of acute liver failure in developed countries is acetaminophen (APAP) overdose (60). In patients with acute liver failure, and in mouse models of liver injury induced by APAP, elevated serum levels of miR-223 have been observed (61, 62). In APAP-induced liver injury, damaged hepatocytes release mitochondria DNA (mtDNA) which activates neutrophils by binding to TLR9. TLR9 then up-regulates miR-223 by enhancing NF-κB binding on the miR-223 promoter. MiR-223 subsequently acts as a negative feedback loop to control APAP-induced liver inflammation by targeting IKKα in neutrophils (62) (**Figure 3**). Most recently, Zhao et al. demonstrated that mice with hepatocyte-specific deficiency of B-cell receptor-associated protein 31 (BAP31-LKO) are more susceptible to APAP-induced hepatotoxicity (63). This model also exhibits reduced stability of factor erythroid 2-related factor 2 (Nrf2) mRNA and reduced miR-223 expression. Nrf2 is an essential transcription factor that mediates cellular anti-oxidative response in liver tissue. Since miR-223 can potentially upregulate Nrf2 protein levels by targeting kelch-like ECH-associated protein 1 (Keap1) in HepG2 cells (64), Zhao et al. have hypothesized that reduced miR-223 expression in liver tissue of BAP31-LKO mice contributes to reduce activation of Nrf2 signaling *via* an increase in Keap1 (63).

In concanavalin A-induced liver injury, miR-223 inhibits IL-1β production by suppressing inflammasome AIM2 in Kupffer cells in the early stage of acute liver failure (65). Surprisingly, during Fas-induced hepatocyte damage, miR-223 deficiency protects against hepatocyte apoptosis and liver injury by targeting the transmembrane tyrosine kinase receptor, insulin-like growth factor 1 receptor (66) (**Figure 3**).

### MiR-223 in Alcoholic Liver Disease

**Figure 4:**
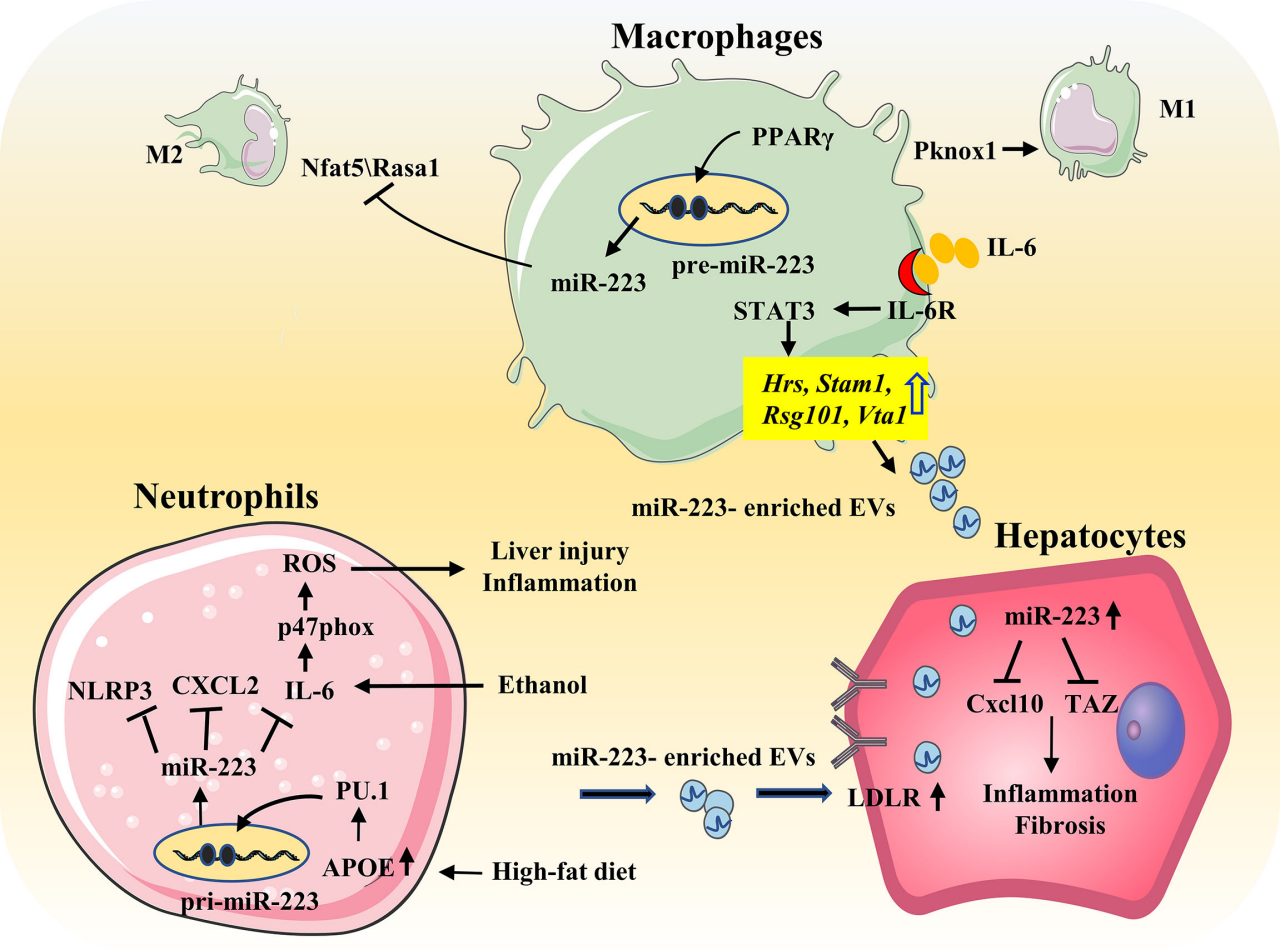
Role of miR-223 in the progression of NAFLD and ALD. During HFD, free fatty acids elevate miR-223 expression in neutrophils by regulating APOE/PU.1 signaling. MiR-223 directly targets NLRP3, CXCL2, IL-6 to ameliorate inflammatory responses. And miR-223 can also form a feedback loop to prevent NASH progression by promoting the preferential uptake of neutrophil-derived miR-223/APOE-enriched EVs in hepatocytes. IL-6 signaling promotes macrophages to release miR-223-enriched exosomes that inhibit expression of several miR-223-targeted genes in hepatocytes, thereby attenuating NASH-associated liver fibrosis. In addition, PPARγ/miR-223 axis control macrophage polarization and protects against diet-induced adipose tissue inflammatory response and systemic insulin resistance. PPARγ can enhance miR-223 expression by directly binding to pre-miR-223 promoter. MiR-223 is required for PPARγ-induced M2 macrophage polarization by controlling expression of the target genes Nfat5 and Rasa1. Moreover, miR-223 inhibits Pknox1 expression, thereby suppressing proinflammatory activation of M1 response. Ethanol elevates miR-223 levels. MiR-223 attenuates neutrophil function by inhibiting the IL-6–p47phox–ROS pathway, thereby protecting against ALD.

ALD represents a spectrum of injury, ranging from simple steatosis to alcoholic hepatitis to cirrhosis. Pathologic progression of ALD is largely driven by inflammatory responses, and it is well-known that hepatic neutrophil infiltration is a hallmark of ALD. Thus, as a neutrophil-specific miRNA, it is hypothesized that miR-223 plays an important role in ALD. Indeed, miR-223 levels are elevated in the serum and/or liver of patients or mouse models with ALD. In chronic-plus-binge ethanol-fed mice, deletion of miR-223 gene exacerbates hepatic neutrophil infiltration, ROS production, and liver injury. Mechanistically, miR-223 inhibits IL-6 expression, and subsequently inhibits p47^phox^ expression in neutrophils, thereby alleviating ethanol-induced hepatic injury and ROS production. However, in alcoholic patients, miR-223 levels in neutrophils have been found to be downregulated, while expression of IL-6 and p47^phox^ are upregulated, compared to healthy controls (26) (**Figure 4**).

A recent study showed that aging increases the susceptibility of alcohol-induced liver injury in both mice and humans *via* downregulation of the SIRT1-C/EBPα-miR-223 axis in neutrophils (13). When circulating neutrophils were obtained from both middle-aged and elderly subjects, levels of SIRT1 and miR-223 were found to be lower than in circulating neutrophils obtained from young individuals. It has been observed that deletion of *Sirt1* gene in myeloid cells exacerbates chronic-plus-binge ethanol-induced liver injury and inflammation, and inhibits miR-223 expression in neutrophils. Mechanistic studies have further revealed that SIRT1 promotes C/EBPα deacetylation by directly interacting with C/EBPα, and subsequently elevated miR-223 levels in neutrophils (13). However, the pathogenetic role of neutrophil infiltration in ALD remains unclear. It is generally believed that neutrophils that infiltrate the liver damage hepatocytes *via* ROS, proteases, and other inflammatory mediators (67); yet in clinical studies of ALD, infiltration of neutrophils is associated with better prognosis. In patients with severe alcoholic hepatitis, neutrophils clear necrotic hepatocytes and secrete hepatocyte growth factor, thereby promoting hepatocyte regeneration and/or controlling bacterial infection (68, 69). For miR-223, it has been shown to contribute to the beneficial roles of neutrophils in ALD.

### MiR-223 in Non-Alcoholic Fatty Liver Disease

The incidence of NAFLD has been increasing each year, and disease progression includes hepatic steatosis, and nonalcoholic steatohepatitis (NASH) which can progress to irreversible cirrhosis, liver failure, and HCC. The pathogenesis of NAFLD is complex and includes metabolic disorders, lipid accumulation, inflammation, oxidative stress, and insulin resistance in hepatocytes (70). MiR-223 plays an important role in the pathophysiology of NAFLD through neutrophils and macrophages (71).

Highly elevated levels of miR-223 been found in the serum and liver of HFD-fed mice and in human NASH samples (48, 72). In addition, miR-223 KO mice are reported to be more susceptible to HFD-induced liver injury, steatosis, inflammation, fibrosis, and HCC. MiR-223 plays a key role in controlling steatosis-to-NASH progression by inhibiting expression of Cxcl10 and transcriptional co-activator with PDZ-binding motif (Taz) in the liver (72). IL-6 signaling in myeloid cells promotes the release of miR-223-rich exosomes from both macrophages and neutrophils. Translocation of these miR-223-rich exosomes into hepatocytes, reduces TAZ (a pro-fibrotic gene in hepatocytes) expression and attenuates NAFLD-associated fibrosis (48). Free fatty acids also enhance the preferential uptake of miR-223-enriched extracellular EVs by hepatocytes. This uptake is dependent on LDLR on hepatocytes and APOE on neutrophil-derived EVs (47) (**Figure 4**). As mentioned above, miR-223 is an important regulator in macrophage polarization, and miR-223 may affect NAFLD progression by inducing a phenotype switch of M2 macrophages (11).

In NAFLD patients, miR-223 levels are increased with the release of miR-223-rich exosomes from neutrophils and macrophages. MiR-223 has been shown to play a role in cellular communication in NAFLD and regulates miR-223 levels in hepatocytes. Thus, miR-223 has the potential to serve as a biomarker for diagnosis and treatment of NAFLD.

### MiR-223 in Liver Fibrosis

Liver fibrosis is the outcome of chronic damage and inflammation caused by various factors such as viral infection, alcohol consumption and non-alcoholic steatohepatitis. In recent years, immune cells, especially macrophages, have increasingly become an active area of research in regard to liver fibrosis. Inflammatory Ly6C^hi^ macrophages and restorative Ly6C^low^ macrophages acts as drivers or inhibitors of liver fibrosis, respectively. Consequently, it is hypothesized that macrophages play a key role in fibrosis (73), and that inflammatory Ly6C^hi^ monocytes could differentiate into Ly6C^low^ macrophages at the site of injury (74).

Considering that miR-223 plays an important role in monocyte/macrophage differentiation, it may also contribute to the pathology of liver fibrosis. In a recent study of the CCl_4_ mouse model, neutrophil-derived miR-223 was found to promote silencing of NLRP3 in proinflammatory macrophages and to induce alternative activation of these macrophages to achieve a restorative phenotype after cessation of injury. Subsequently, production of IL-10 by the restorative macrophages can indirectly reduce inflammation and early fibrosis by suppressing hepatic stellate cell activation and ameliorating collagen formation and deposition (32).

In HCV-positive cirrhosis, serum miR-223 is dysregulated (75). Meanwhile, in HBV-related liver fibrosis, serum miR-223 is progressively downregulated from S0-S2 (early fibrosis) to S3-S4 (late fibrosis) (76). However, it has also been reported that serum miR-223 is upregulated in significant fibrosis (≥F2) compared with no/mild fibrosis (F0-F1); and is upregulated in severe fibrosis (≥F3) and cirrhosis (F4) compared with F0-F2 and F0-F3, respectively (77). Therefore, further studies are needed to evaluate the potential for miR-223 to serve as a noninvasive biomarker of fibrosis progression.

### MiR-223 in HCC

MiR-223 is commonly repressed in human HCC, partly due to epigenetic regulation by sulfatide (17, 78). MiR-223 inhibits tumor cell proliferation and promotes apoptosis *via* inhibition of the mTOR pathway by targeting Rab1 or by targeting NLRP3 (as observed in several HCC cell lines) (79, 80). In patients with HBV-related HCC, miR-223 was found to be significantly reduced in cancerous tissues compared with non-cancerous tissues, and it also exhibited a negative correlation with tumor size and Barcelona Clinic Liver Cancer stage. Therefore, circulating miRNA-223-3p may represent a novel diagnostic and prognostic marker for patients with HBV-associated HCC (81).

There are several carcinogenesis genes which have been identified as potential targets of miR-223 and contributors to the anti-HCC effect of miR-223. For example, stathmin1 is a target gene of miR-223 which is overexpressed in HCC. Functional inhibition of stathmin1 has decreased cell viability and proliferation, while increasing apoptosis in HCC cells (78). Microarray analyses of hepatic gene expression in HFD-fed miR-223 KO mice have also revealed that the most dysregulated genes are cancer-related, and this likely contributes to the increased susceptibility of miR-223 KO mice to HFD-induced liver cancer (72).

**Figure 5:**
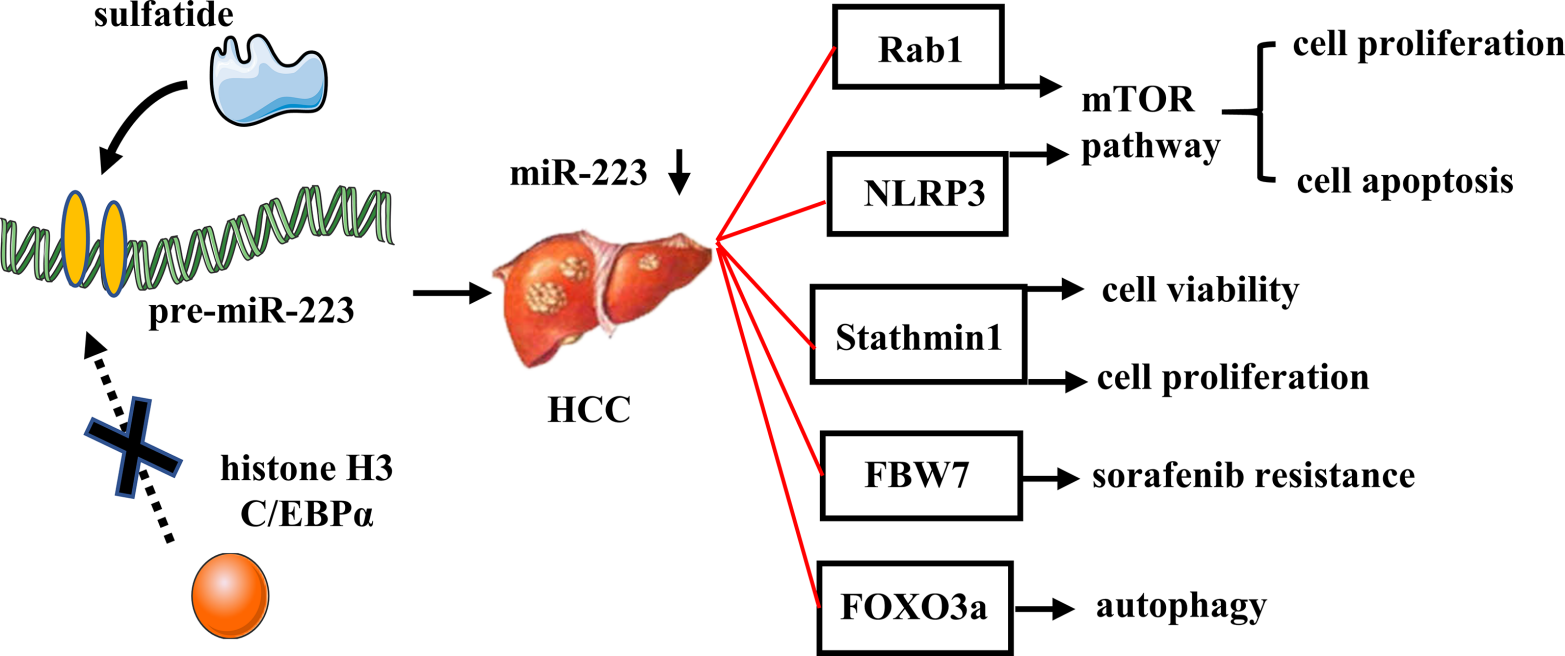
Role of miR-223 in HCC. In HCC, sulfatide acts on the promoter region of pre-miR-223, thereby reducing the recruitment of histone H3 with C/EBPα and decreasing miR-223 expression. MiR-223 directly targets Rab1, NLRP3, Stathmin1, FBW7, and FOXO3a, which involved in tumor cell proliferation, apoptosis, autophagy, and drug resistance.

Patients with HCC usually acquire resistance over the course of long-term chemotherapy, and this can severely compromise the therapeutic benefits of this treatment approach. To date, the role of miR-223 in chemoresistance remains contradictory. For example, miR−223 expression is increased in sorafenib−resistant HCC cells, and knockdown of miR−223 markedly enhances the sensitivity of HCC cells to sorafenib. The latter involves an increase in expression of miR-223 target genes, F-box and WD repeat domain-containing 7 (FBW7), suggesting that miR-223 may represent a potential target for overcoming sorafenib resistance (82). However, it has also been reported that miR-223 is expressed at low levels in doxorubicin-treated HCC cells. It is possible that overexpression of miR-223 can inhibit doxorubicin-induced autophagy, thereby increasing doxorubicin cytotoxicity in HCC cells. Moreover, miR-223 has been shown to inhibit excessive autophagy in HCC cells by targeting FOXO3a (83). **Figure 5** and **Table 1** summarized the role of miR-223 in HCC.

**Table 1:** MiR-223 expression, target, and function in liver diseases.

Liver disease	miR-223 expression		target	Clinical usage/function	Reference
	Circulating	Cellular/hepatic			
**Viral hepatitis**					
Chronic hepatitis C	↑	–	–	Regulate the infection process of HCV	(58)
Chronic hepatitis B	↓	–	–	As a new marker for hepatitis B diagnosis	(56)
HIV with HCV	–	↑	MX1, IFI27, CD169	Inhibition of inflammatory NF- κB pathway	(59)
**Acute liver injury**					
Mouse model of FFC	↑	↑	NLRP3, IL-1β	Suppress the activation of the NLRP3 inflammasome	(84)
Fas-Induced in miR-223 KO mouse	–	↓	IGF-1R	MiR-223 deficiency protects against Fas-induced hepatocyte apoptosis and liver injury	(66)
Mouse model of CCl_4_ or BDL-induced liver injury	–	↑	–	MiR-223 represents a promising diagnostic marker in a panel of serum markers of liver injury	(61)
High glucose-induced T2DM HepG2 cells	–	↑	keap1	MiR-233 regulates oxidative stress by targeting keap1-Nrf2 system to affect the pathological process of liver injury in T2DM	(64)
Mouse model of APAP-induced DILI	–	↑	IKKα	Up-regulation of miR-223 in terminating the acute neutrophilic response	(62)
**ALD**					
SIRT1 KO mice	–	↓	SIRT1	Increases the susceptibility of alcohol-induced liver injury in mice and humans through the down-regulation of the neutrophilic SIRT1-C/EBPα-miR-223 axis	(13)
Chronic-plus-binge ethanol feeding mouse model	↑	↑	IL-6	MiR-223 directly inhibited IL-6 expression and subsequently inhibited p47phox expression in neutrophils	(26)
**NAFLD**					
HFD-fed miR-223 KO mice	–	↑	Cxcl10, Taz	MiR-223 controls steatosis-to-NASH progression by inhibiting hepatic Cxcl10 and Taz expression	(72)
IL-6 KO mice	–	↑	IL-6	IL-6 signaling promotes microRNA-223-enriched exosome production to attenuate NAFLD-associated fibrosis	(48)
HFD-fed miR-223KO mice	–	↑	–	LDLR and APOE in the selective control of miR-223-enriched EV transfer from neutrophils to hepatocytes	(47)
Mouse model of CFD diet caused NFALD	–	↑	IRP1	Deregulation of hepatic iron homeostasis	(85)
**Liver fibrosis**					
HBV-related liver fibrosis	↓	–	–	Serum miRNA-223 levels could serve as a potential noninvasive biomarker of fibrosis progression	(76)
HCV-related liver fibrosis	↑	–	–	Predicting the progression of hepatic fibrosis in patients with hepatitis C	(77)
CCl_4_ mouse model	–	↑	–	Hepatic neutrophils as resolving effector cells that induce proinflammatory macrophages into a restorative phenotype, potentially *via* miR-223	(32)
**HCC**					
HBV-related HCC	–	↓	–	New diagnostic and prognostic markers representing patients with HBV related HCC	(81, 86)
HCC cell lines	↓	↓	Rab1, NLRP3	Inhibit tumor cell proliferation and promote apoptosis	(79, 80)
hepatocellular carcinoma cells	–	↓	FOXO3a, FBXW7	Promote drug resistance of hepatocytes	(82, 83)
HCC patients	↓	↓	Stathmin1	MiR-223 is down-regulated in the development of HCC	(78)

↑ increase; ↓ decrease.

### MiR-223-Based Therapies

MiR-223 regulates multifarious biological functions in the pathogenesis of liver diseases, thereby making it an attractive therapeutic target. As such, it has been tested in various preclinical models of liver diseases. For example, a synthetic miR-223 analog, miR-223-3p, has been used to treat acute and chronic hepatitis by silencing activation of the NLRP3 inflammasome. It has also been demonstrated that miR-223-3p reduces the infiltration of neutrophils, monocytes, and early activated macrophages, and it suppresses transcriptional expression of pro-inflammatory cytokines (IL-6 and IL-12) and chemokines (Ccl2, Ccl3, Cxcl1, and Cxcl2) in lipopolysaccharide (LPS)/D-GalN-induced endotoxin acute hepatitis. MiR-223 3p treatment inhibited the activation of hepatic stellate cells and ameliorated fibrosis development in mice with fibrotic NASH. Furthermore, miR-223-3p negatively regulates activation of the NLRP3 inflammasome by downregulating expression of NLRP3, IL-1β, and activation of caspase-1 in both endotoxin acute hepatitis and fibrotic NASH (84).

As mentioned above, EVs are highly stable, less toxic, and are preferentially taken up by the liver. Thus, EVs represent an attractive delivery vehicle for miRNA-based therapies in liver diseases. Indeed, fibroblast-derived EVs have been used as vehicles to deliver miR-195 to cancer cells and reduce tumor volume, and have also improved survival in a rat model of cholangiocarcinoma (87). Meanwhile, hepatic stellate cell-derived EVs loaded with miR-335-5p have decreased HCC growth and invasion both *in vitro* and *in vivo* (88). Therefore, the potential for EVs to deliver miR-223 to treat liver diseases appears to be promising, although identification and characterization of the factors that mediate EV stabilization and uptake is still needed.

## Conclusions and Prospects

In summary, miR-223 plays an important role in cell differentiation and inflammatory response. MiR-223 is also involved in regulating several processes in myeloid differentiation, and activation of neutrophils and macrophages. Many studies have shown that miR-223 expression is dysregulated in liver physiology and pathology, and dysregulated miR-223 is closely associated with viral hepatitis, liver inflammation, fibrosis, steatosis, and HCC. However, miR-223-mediated mechanisms among the various liver diseases remain to be elucidated. For example, in viral hepatitis, the accurate role of miR-223 in viral infection is still not well understood, although it seems to act as a negative regulator of immune regulation and inflammatory response during viral infection. Yet the mechanistic details about regulation of virus-induced inflammation by miR-223 remain to be characterized.

Dysregulation of miR-223 has been reported in liver diseases which can be modified by miRNA mimics or anti-miRNAs. As such, miR-223 has a potential to serve as a promising biomarker and therapeutic strategy for the treatment of liver diseases. However, circulating miR-223 is differentially expressed in different periods of liver diseases, and even in the same disease. For example, in HBV-related HCC, circulating miR-223 levels was differently regulated that reported by different studies. Thus, large-sample clinical studies are required to further determine its diagnostic value in liver pathologies. It is also important to point out that miR-223-based studies have mostly been conducted with mouse models. However, there is a great need for clinical studies to be conducted in order to further evaluate the effectiveness of miR-223 against specific liver diseases. Furthermore, miR-223-based therapy should consider the cell-specific and disease-specific functions of miR-223 in the liver, which will likely help us to increase the therapeutic potential and avoid off-target effects. A combination of bioinformatic-based predictions to identify miR-223 target genes and additional studies of the role of miR-223 in inflammation and cancer could provide a better understanding of miR-223 biology, and facilitate the development of safe, accurate, and specific therapies for liver diseases.

## Author Contributions

JG and XH wrote the manuscript. HX and YC collected literatures. NL revised the article. All authors contributed to the article and approved the submitted version.

## Funding

This work was supported by the National Natural Science Foundation of China (No. 82170603) and the Natural Science Foundation of Zhejiang Province (No. LY22H030006).

## Conflict of Interest

The authors declare that the research was conducted in the absence of any commercial or financial relationships that could be construed as a potential conflict of interest.

## Publisher’s Note

All claims expressed in this article are solely those of the authors and do not necessarily represent those of their affiliated organizations, or those of the publisher, the editors and the reviewers. Any product that may be evaluated in this article, or claim that may be made by its manufacturer, is not guaranteed or endorsed by the publisher.
